# Two new genera and three new species of cavernicolous trechines from the western Wuling Mountains, China (Coleoptera, Carabidae, Trechinae)

**DOI:** 10.3897/zookeys.1059.70009

**Published:** 2021-09-03

**Authors:** Mingyi Tian, Sunbin Huang, Xinyang Jia, Yi Zhao

**Affiliations:** 1 Department of Entomology, College of Plant Protection, South China Agricultural University, 483 Wushan Road, Guangzhou, 510642, China South China Agricultural University Guangzhou China; 2 Mécanismes adaptatifs et évolution (MECADEV), UMR 7179 CNRS–MNHN, Muséum national d’Histoire naturelle, CP50, 57 Rue Cuvier, 75005 Paris, France Muséum national d’Histoire naturelle Paris France

**Keywords:** Aphaenopsian, cave-dwelling, Chongqing, ground beetles, Guizhou, semi-aphaenopsian

## Abstract

Two new genera and three new species of cave-adapted ground beetles belonging to the tribe Trechini are established and described: *Wulongiusqilinger***gen. nov.** and **sp. nov.** from limestone cave Qiankou Dong (Chongqing: Wulong), *Qianotrechuscongcongae***sp. nov.** from cave Shigao Dong (Chongqing: Nanchuan), and *Qianlongiuszhoui***gen. nov.** and **sp. nov.** from cave Qianlong Dong (Guizhou: Songtao). *Wulongiusqilinger***sp. nov.** is a small aphaenopsian beetle with a thin and elongated body, while *Qianlongiuszhoui***sp. nov.** is a semi-aphaenopsian with a stout body. Both new genera are not closely related to any genus of Trechini occurring in the South China Karst, and so their systematic positions remain unclear.

## Introduction

**Figure 1.**
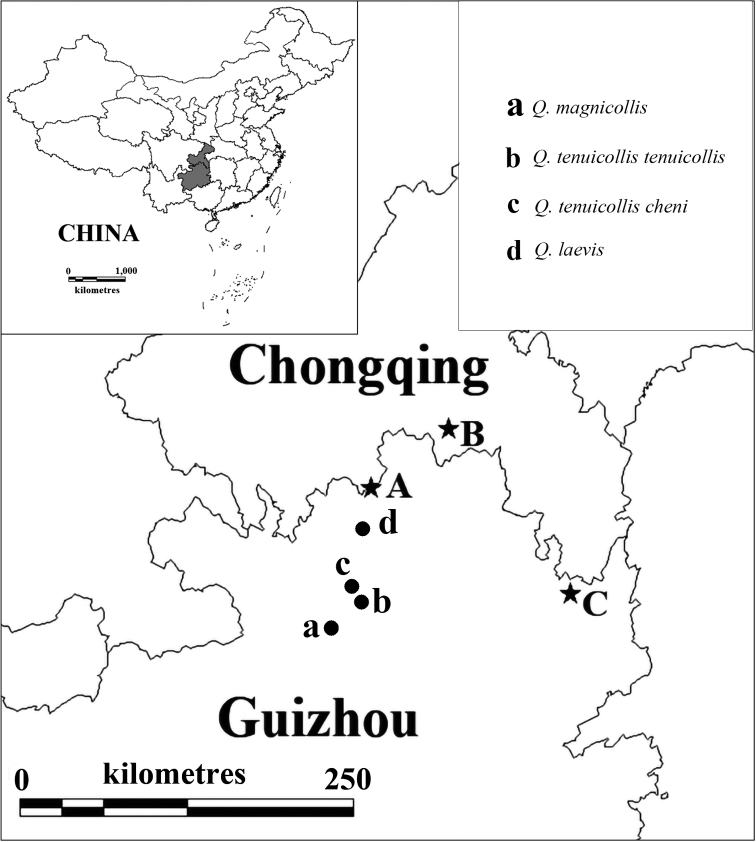
A distribution map showing the localities of the three surveyed caves (stars) and the known species and subspecies of the genus *Qianotrechus* Uéno, 2000 (dots) **A** Shigao Dong **B** Qiankou Dong **C** Qianlong Dong.

The scientific discoveries made in recent years have revealed that the Wuling Mountains harbour a very rich fauna of cavernicolous trechine beetles which is composed of over 30 species in 13 genera (Tian et al. 2021). However, our knowledge on cave beetles in these regions is still increasing. In April 2021, we conducted a biological survey in three limestone caves in the eastern part of the Wuling Mountains in Chongqing Shi and Guizhou Province (Fig. 1). The main purpose of our survey was to investigate the cave biodiversity in the Furong Dong cave system, a well-known show cave in Wulong, Chongqing, which is also a World Heritage Site of South China Karst. Thanks to the assistance of several cavers from the Qilinger Cave Exploration Team (Nanning) and Chongqing Cave Exploration Team (Chongqing), we could successfully survey in cave Qiankou Dong, an upper cave of the Furong Dong system in Tianxing Karst, Wulong. In the deepest point of the vertical pit, which is 55 m deep, we came across a single beetle. This strange looking trechine was undoubtedly an aphaenopsian due to its thin body and elongated appendages. Further study in the laboratory showed that it is a representative of an unknown lineage of cave trechine beetles in China. On the way to Wulong, we visited a cave named Shigao Dong in Nanchuan, Chongqing, and collected two trechine individuals. They are members of a new species belonging to the genus *Qianotrechus* Uéno, 2000 and close to *Q.laevis* Uéno, 2000. After the survey in Wulong, we had an opportunity to investigate Qianlong Dong, a beautiful show cave in Songtao Maio Autonomous County, northeastern Guizhou, thanks to the assistance of Mr Wenlong Zhou (Guizhou Institute of Mountainous Region Resources, Guizhou Academy of Sciences, Guiyang), and discoveried a new species of semi-aphaenopsian trechine, which is also a representative of an unknown genus. In this paper, we describe three new species of cavernicolous trechine beetles discovered in caves Qiankou Dong (Wulong, Chongqing), Qianlong Dong (Songtao, Guizhou) and Shigao Dong (Nanchuan, Chongqing). We also establish two new genera to place the first and second new species.

## Material and methods

All beetles for this study were collected with the naked eyes using an aspirator in dark zones of the caves and kept in vials with 50% ethanol before studying. One exemplar of each species (but three legs removed from the holotype of *Wulongiusqilinger* gen. nov. and sp. nov.) were kept into 95% ethanol for DNA sequencing. Dissections and observations were made by using a binocular Leica MZ75 dissecting microscope (Wetzlar, Germany). Dissected genitalia, including the median lobe and parameres of the aedeagus, were glued on small transparent plastic cards and pinned under the specimen from which they were removed. Digital pictures were taken using a Canon EOS 5D Mark III camera (Tokyo, Japan) and then processed by means of Adobe Photoshop CS5 software (Adobe System Incorporated, California, USA). Measurements and terminologies used in the text are as in Tian et al. (2016). All specimens of the type series are deposited in the insect collection of South China Agricultural University, Guangzhou, China (SCAU).

## Taxonomy

### Wulongius

Taxon classificationAnimaliaColeopteraCarabidae

Tian & Huang gen. nov.

2CD9F68D-CA88-550C-8504-AFBFB2E0D39F

http://zoobank.org/0274541E-3999-4F57-8016-491DBC9422EA

#### Type species.

*Wulongiusqilinger* Tian & Huang, sp. nov. (cave Qiankou Dong, Wulong, Chongqing).

#### Generic characteristics.

Medium-sized for cavernicolous trechine beetles, aphaenopsian and depigmented; body thin and elongate, with thin and slender appendages. Head glabrous, pronotum covered with dense and long erected setae; apical portions of elytra covered with very sparse and short hairs; head strongly elongate, much longer than wide (excluding mandibles); widest at about middle, gently narrowed posteriad, neck constriction short; 2 pairs of supra-orbital setiferous pores present; frontal furrows short and incomplete, ending at the level of the head widest portion; vertex strongly convex; mandibles thin, sharply hooked at apices, right mandibular tooth completely reduced; labial suture clear; mentum with 2 setae on each side of median tooth, base of mentum with small basal pits on each side; submentum with a row of 10 setae; palps thin and very elongate, all glabrous but bisetose on inner margin of 2^nd^ labial palpomere; antennae very thin and long, extending over apices of elytra. Propleura visible from above; pronotum cylindrical, distinctly elongated, much longer than wide, lateral margins nearly parallel-sided though slightly divergent medially, presence of only anterior latero-marginal setae (posterior ones lacking), both fore and hind angles widely obtuse. Scutellum present. Elytra elongated ovate, dorsum strongly convex and expanded laterally, partly concealing lateral margin of elytra in middle portion; humeral angle indistinct, lateral margin well bordered and ciliate throughout; striae almost obsolete though traceable; 2 pairs of dorsal setiferous pores present on the 3^rd^ stria, preapical pores present; basal pores located behind scutellum; the humeral group of the marginal umbilicate pores not aggregated, 1^st^ pore inwardly and backwardly shifted to the site of 6^th^ stria and located at level between 2^nd^ and 3^rd^ pores, 5^th^ and 6^th^ pores widely spaced each other.

#### Remarks.

The position of *Wulongius* gen. nov. within Trechini is undetermined. It might be related to the genus *Xiangxius* Tian & X. Huang, 2021, which has been recently described from Tangle Dong cave in western Hunan, in the eastern Wuling Mountains. Both genera are aphaenopsian, with highly specialized morphological characters. They are somewhat similar in thoracic configuration and, in particular, in the elytral chaetotaxy, in which the 1^st^ pore of the marginal umbilicate series is backwardly and inwardly shifted and both 5^th^ and 6^th^ pores are widely spaced. However, there are many different important characters between them including the body shape: (1) elytra are much more convex and expanded laterally in *Wulongius*, concealing median part of the lateral margins, and without aprotruding humeral angle on each elytron, versus elytra less expanded laterally in *Xiangxius*, whole lateral margin visible from above, and with a distinct protruding humeral angle on each elytron; (2) pronotum covered with long setae, without posterior latero-marginal setae in *Wulongius*, versus pronotum glabrous, with posterior latero-marginal setae in *Xiangxius*; (3) mentum glabrous in *Wulongius*, but pubescent in *Xiangxius*; (4) labial suture clear in *Wulongius*, whereas mentum fused with submentum in *Xiangxius*; (5) right mandibular tooth edentate in *Wulongius*, while bidentate in *Xiangxius*; (6) submentum with a row of 10 setae in *Wulongius*, instead of 15 or 16 setae in *Xiangxius*; (7) head slender in *Wulongius*, not thickened, with longer antennae extending beyond the apices of elytra, versus head stout, widely convex laterally, and with shorter antennae extending only to apical 2/3 of elytra in *Xiangxius*.

#### Etymology.

“*Wulong*”+ “-*ius*”, indicating the homeland of this new genus. Gender masculine.

#### Generic range.

China (Chongqing). Known only from limestone cave Qiankou Dong (Fig. 1).

### Wulongius qilinger

Taxon classificationAnimaliaColeopteraCarabidae

Tian & Huang sp. nov.

EDAF73B8-7D89-5775-A147-1975A66E1735

http://zoobank.org/FE36775A-E68D-47FB-8309-ED3999B229FB

**Figure 2.**
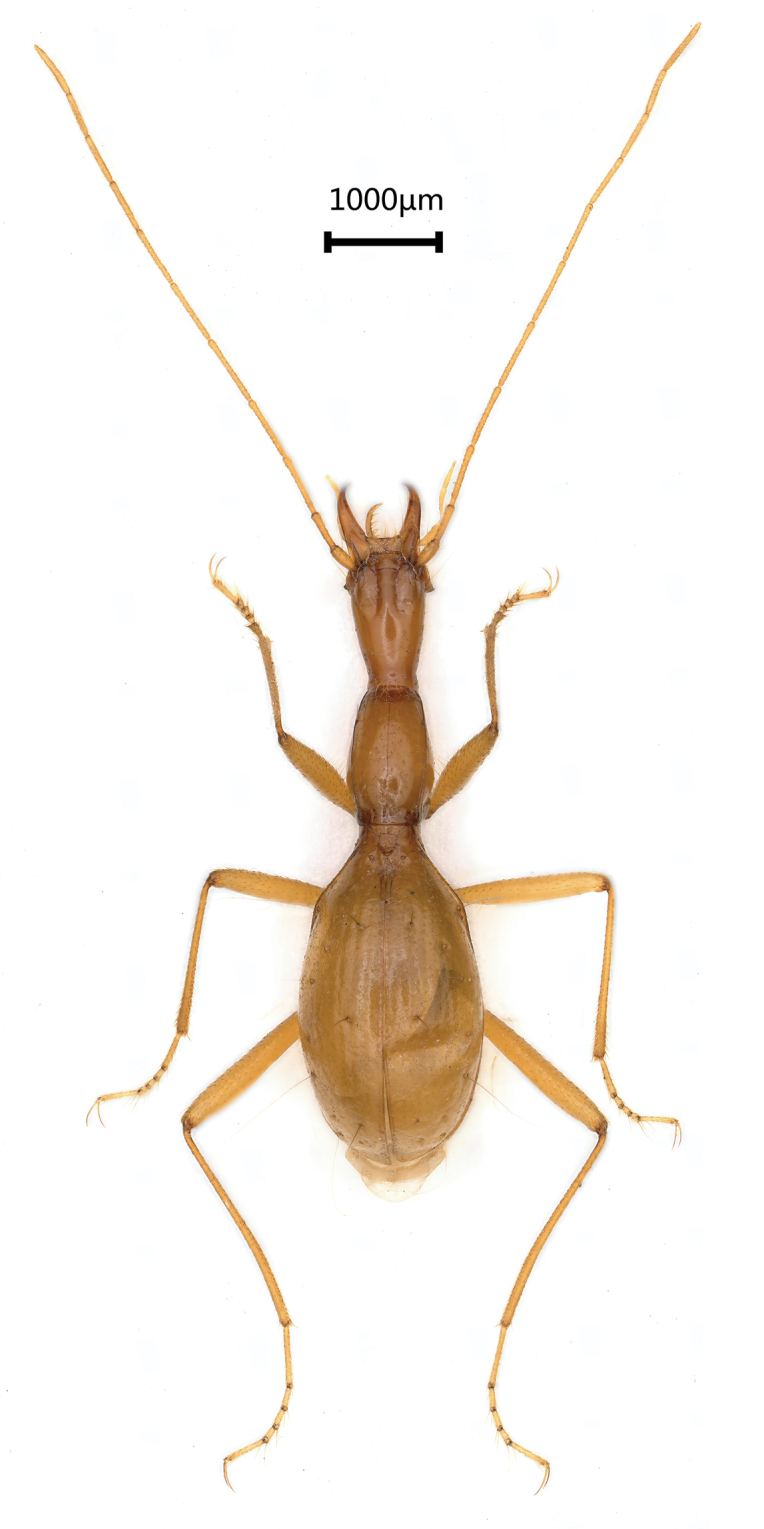
*Wulongiusqilinger* gen. nov. and sp. nov., habitus, holotype female.

**Figure 3.**
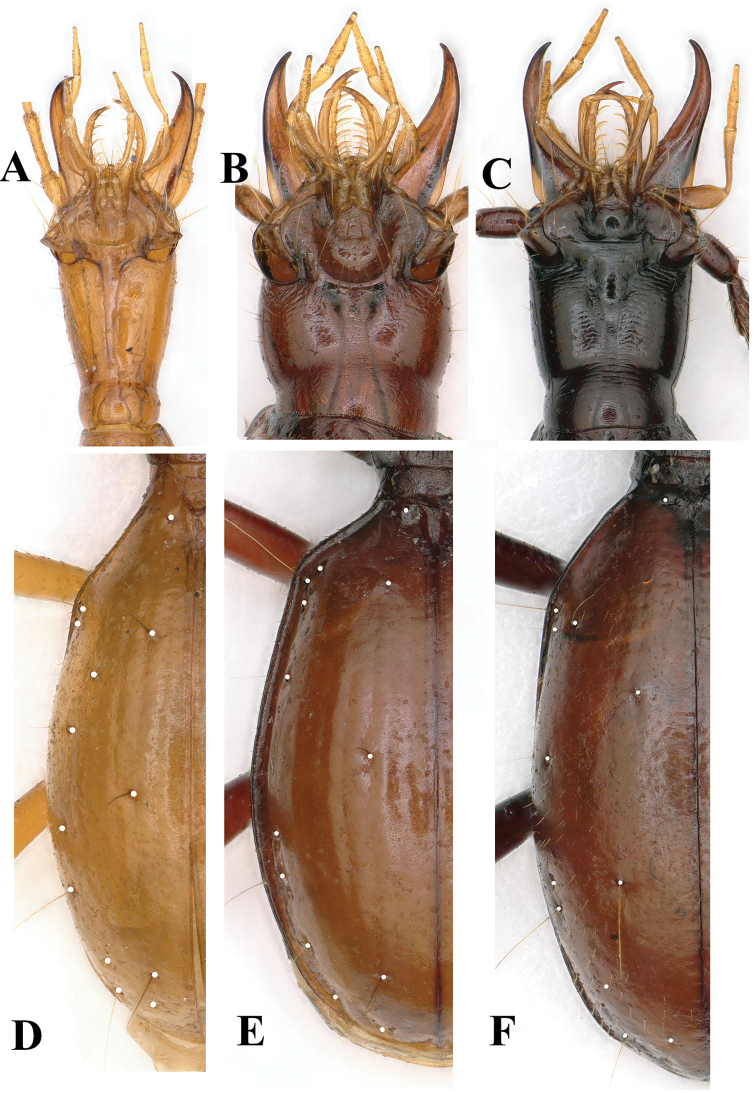
Ventral head (**A–C**) and elytral chaetotaxy (**E, F**) of cave beetles **A, D***Wulongiusqilinger* gen. nov. and sp. nov. **B, E***Qianotrechuscongcongae* sp. nov. **C, F***Qianlongiuszhoui* gen. nov. and sp. nov.).

**Figure 4.**
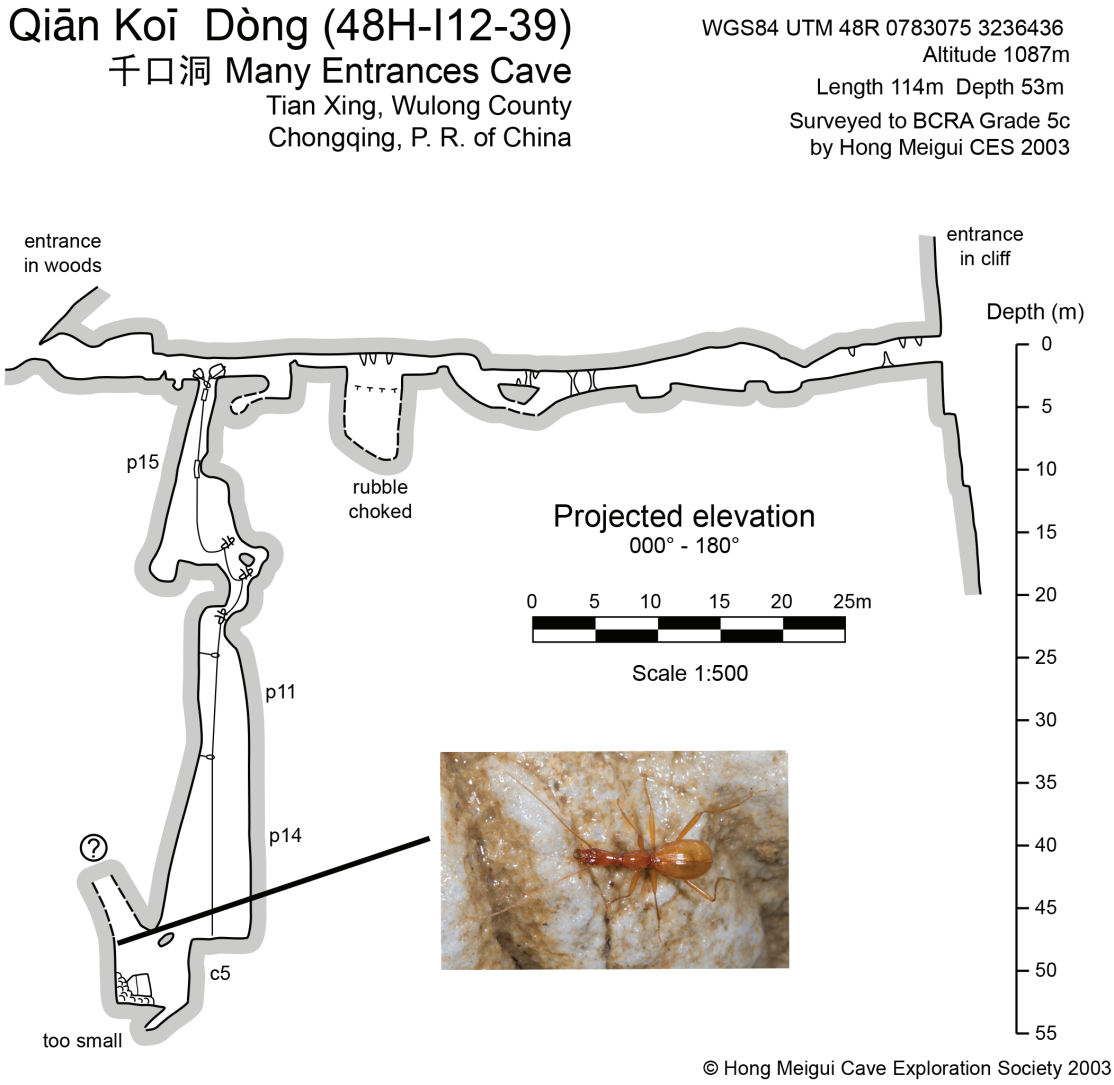
Map of Qiankou Dong, to show where the holotype of *Wulongiusqilinger* gen. nov. and sp. nov. was discovered) (courtesy of Prof. Yuanhai Zhang and Hong Meigui Cave Exploration Society).

**Figure 5.**
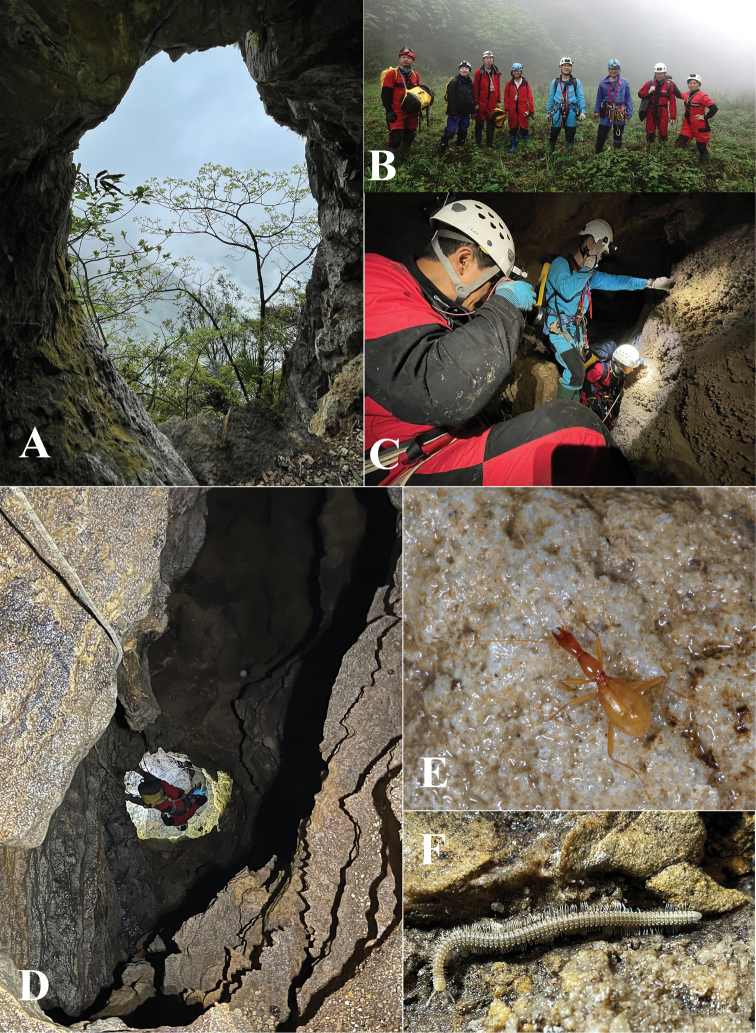
Cave Qiankou Dong **A** entrance in the cliff **B** team photo outside the cave **C** surveying in the cave **D** the vertical pit **E** a running individual of *Wulongiusqilinger* gen. nov. and sp. nov. **F** a millipede, *Glyphiulus* sp. (team photo courtesy of Mr Lixin Chen).

Figures 1 , 2 , 3 , 4 , 5

#### Type material.

***Holotype*** female, cave Qiankou Dong, Tongluo, Jiangkou, Wulong, Chongqing, 29.32°N, 107.91°E, 1103 m, 2021-IV-14, leg. “Wu Ya” (a nickmane of Mr Hongying Wu) & Mingyi Tian, in SCAU.

#### Diagnosis.

A medium-sized aphaenopsian trechine species, with a distinctly elongated body and thin appendages, without eyes and pigmentation, elytra remarkably expanded laterally and partially concealing median part of elytral margins.

#### Description.

Length: 6.5 mm, width: 1.6 mm. Habitus as in Figure 2. Body yellow, antennae, palps and tarsi pale; head covered with a few sparse setae, pronotum covered with dense and long setae, elytra smooth and glabrous in most parts, but with short and sparse setae along lateral margins, underside of head with a few setae laterally and ventrally, sparsely setose on ventral thorax and abdominal ventrites medially; moderately shining. Microsculpture reduced on head; striated on pronotum and elytra.

Head much longer than wide, HLm/HW = 2.68, HLl/HW = 1.96; genae not expanded, widest at about middle of head excluding mandibles, gradually narrowed posteriad, neck short and narrow; frons and vertex moderately convex; frontal furrows nearly parallel-sided, shortly divergent apically, ending at about middle of head; anterior and posterior supraorbital pores located at about basal 3/8 and 1/5 of head, respectively; clypeus 4-setose; labrum transverse, frontal margin almost straight, 6-setose; mentum bisetose, tooth rather narrow, bifid at tip, slightly longer than half of the lateral lobes; ligula 10-setose at apex, inner 2 much longer than others; distal palpomeres of maxilla and labium 1.3 times as long as the penultimate palpomeres; suborbital pores much closer to neck constriction than to submentum (Fig. 3A); antennae pubescent from pedicle to 11^th^ antennomeres, scape not pubescent, stouter and shorter than other articles, with several long setae; relative length of each antennomere compared with scape as follows: the 1^st^ (1.0), 2^nd^ (1.4), 3^rd^ (1.9), 4^th^ (2.2), 5^th^ (2.5), 6^th^ (2.3), 7^th^ (2.1), 8^th^ (1.8), 9^th^ (1.8), 10^th^ (1.5) and 11^th^ (2.0).

Prothorax distinctly tumid at sides, propleura slightly wider than pronotum, PrW/PnW = 1.1, visible from above, widest a little before basal 1/3, wider than head, PrW/HW = 1.2; pronotum much longer than wide, PnL /PnW = 1.6, shorter than head excluding mandibles, PL/HL l = 0.9; widest at about 3/7 of pronotum from base; lateral margins finely bordered throughout, gently contracted forwards and backwards, slightly sinuate before hind angles which are wide and blunt though more or less rectangular; frontal angles rounded off; base straight, front margin slightly arcuate, both unbordered, base slightly wider than front margin; only anterior latero-marginal setae present, at about 2/9 from front margin; disc slightly convex, mid-line well-marked; both fore and basal transversal impressions faint, basal foveae shallow. Scutellum quite large.

Elytra (Fig. 2, 3D) almost as long as fore body including mandibles, much longer than wide, EL/EW = 1.9, almost twice as wide as prothorax, EW/PrW = 2.1; base unbordered, lateral margins not serrate but ciliate throughout, widest at about middle, humeral angles obtuse, margins invisible from above in middle; apical striole reduced; basal pore present, anterior and posterior dorsal pores on the 3^rd^ stria at basal 2/7 and apical 4/9 of elytra, respectively; preapical pores located at apical anastomosis of 3^rd^ and 4^th^ striae; only an apical pore present, subequal to suture and to margin of elytra; the anguloapical pore absent; only 2^nd^ and 3^rd^ pores of marginal umbilicate group close to the marginal gutter, 1^st^ pore closer to 4^th^ than to 2^nd^; the 7^th^ and 8^th^ pors distant from marginal gutter.

Legs densely pubescent; 1^st^ tarsomere shorter than 2^nd^–4^th^ combined in fore legs, whereas slightly longer and as long as in middle and hind legs, respectively; tibiae without longitudinal sulci.

Ventrite IV–VI bisetose, VII quadrisetose in female.

**Male.** unknown.

#### Etymology.

Referring to the Qilinger Cave Exploration Team (Nanning), led by Mr Lixin Chen, a famous TV reporter on cave exploration in China.

#### Distribution.

China (Chongqing). Known only from limestone cave Qiankou Dong (Fig. 1). Qiankou Dong (Figs 4, 5) is located at about 200 m from the beautiful show cave Furong Dong to the northeast and probably was the upper part of the Furong Dong cave system in the past (Yuanhai Zhang pers. comm.). It was well surveyed by the Hong Meigui Cave Exploration Society (U.K.) in 2003. The cave is 114 m long, with two entrances, one in a forest near a farm house and the other in a cliff. There is a 55 m deep vertical pit, although the passage of the cave is more or less horizontal. The single specimen of the new species was discovered by “Wu Ya” (Mr Hongying Wu, an active and well-known caver, who discovered many Tiankengs in China by using Google Earth) and M. Tian. Other cave animals also observed in Qiankou Dong are millipedes (*Oxidusgracilis* (C.L. Koch, 1847), *Eutrichodesmus* sp., *Epanerchodus* sp., and *Glyphiulus* sp.), a cricket (*Tachycines* sp.), an earwig (*Challiafletcheri* Burr, 1904), spiders (*Parasteatodatepidariorum* (C.L. Koch, 1841) and *Pholcus* sp.), and an ant-loving beetle (*Batrisocenus* sp.).

### Qianotrechus congcongae

Taxon classificationAnimaliaColeopteraCarabidae

Tian & Zhao sp. nov.

74CF9B71-5E20-5704-A730-270742F139CA

http://zoobank.org/51137F01-C791-4B90-B94F-64822C00FD2C

**Figure 6.**
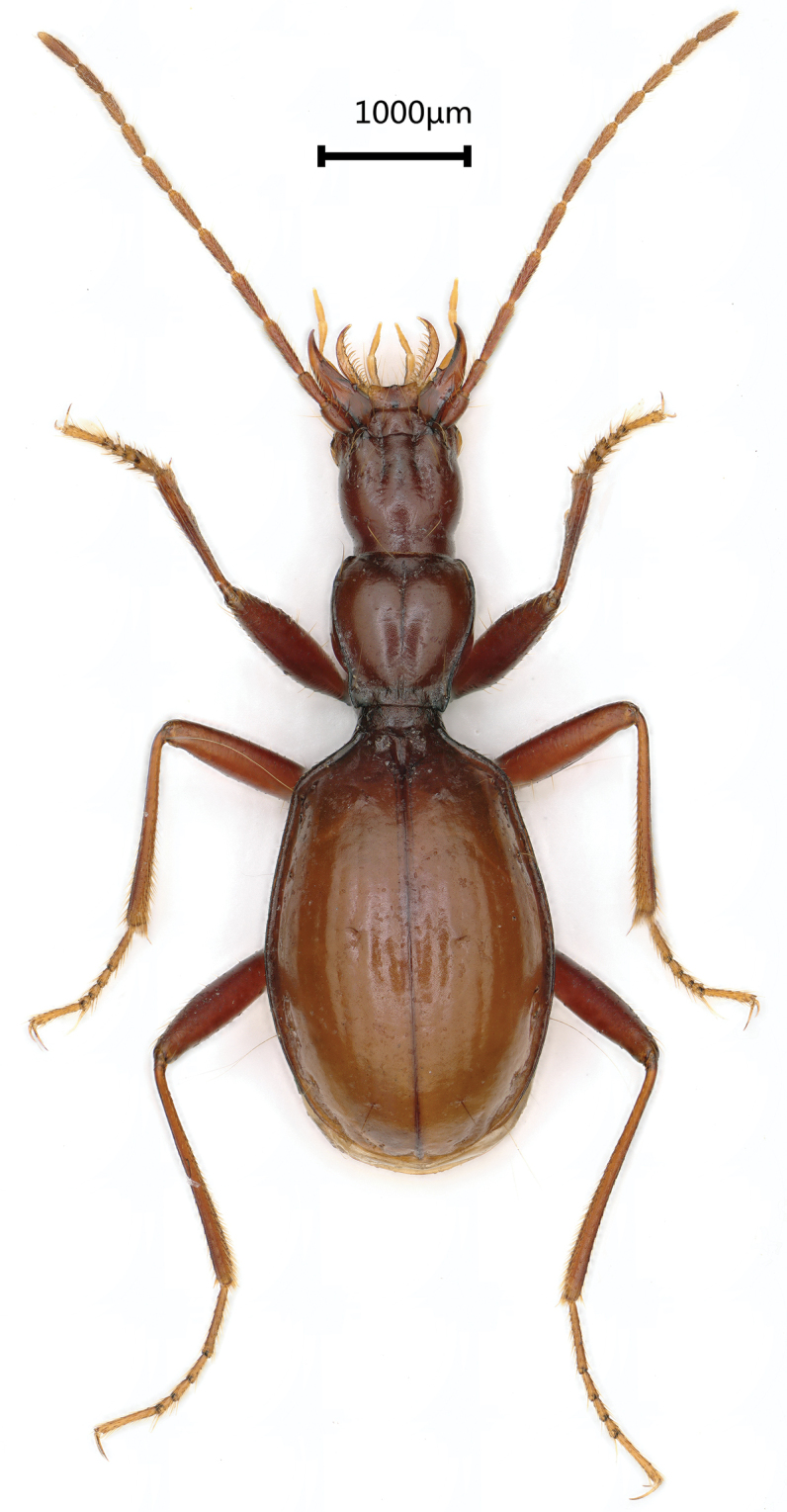
*Qianotrechuscongcongae* sp. nov., habitus, holotype male.

**Figure 7.**
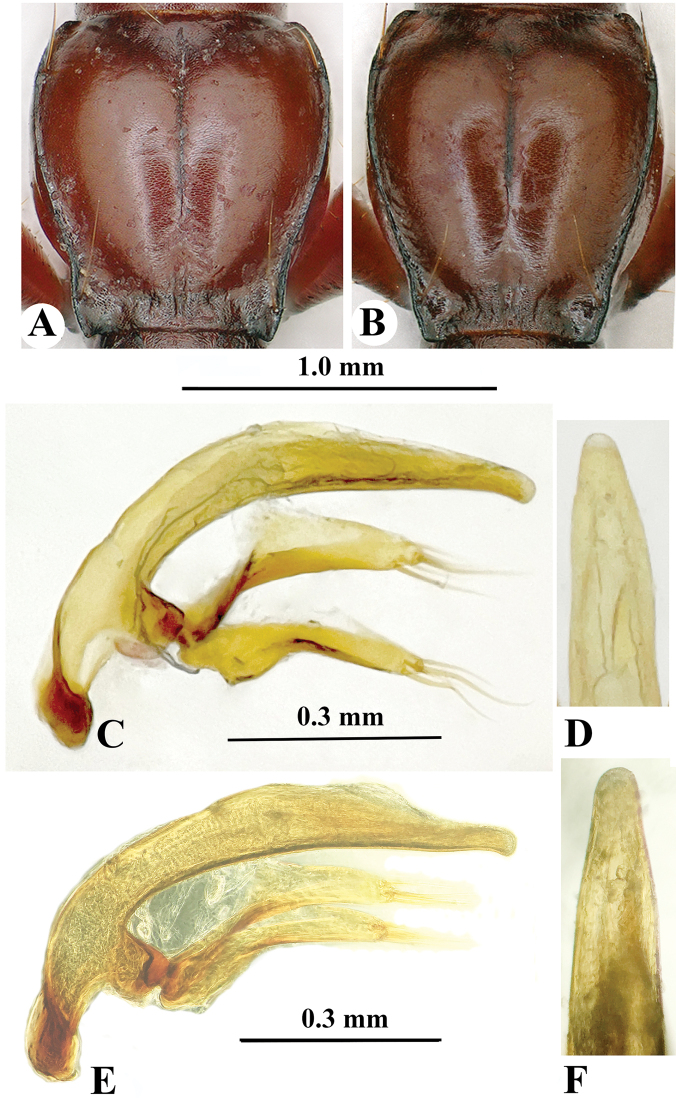
Pronotum and male genitalia of *Qianotrechus* species. **A, C, E***Q.congcongae* sp. nov. **B, D, F***Q.laevis* Uéno, 2000 **A, B** pronotum **C, E** median lobe and parameres, lateral view **D, F** apical lobe, dorsal view.

**Figure 8.**
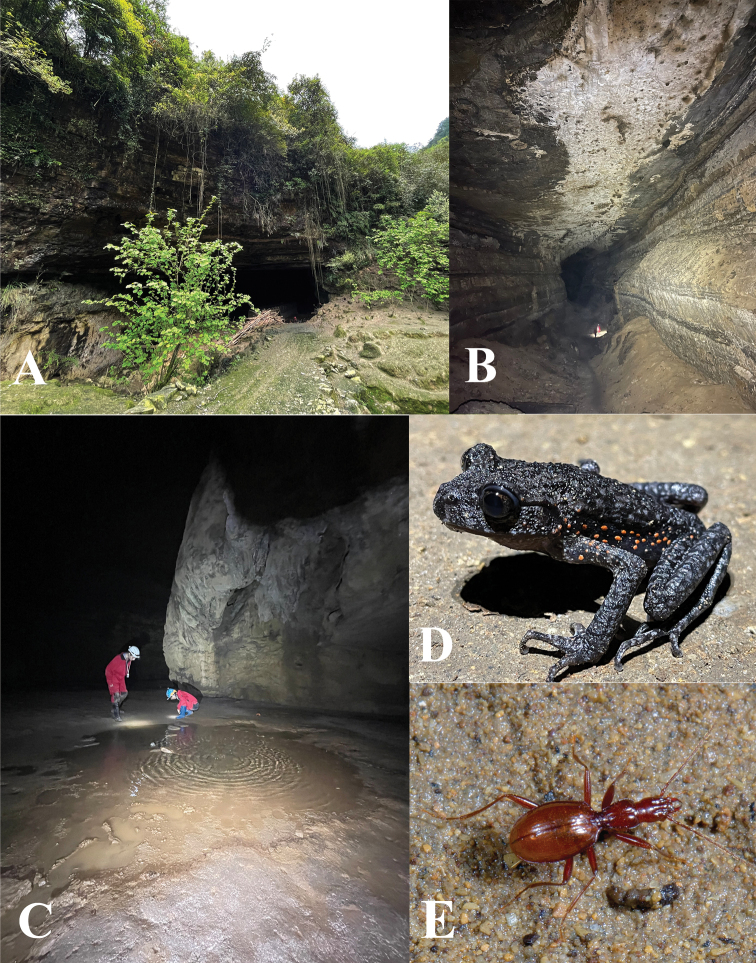
Cave Shigao Dong. **A** entrance **B** the huge passage **C** place where the two beetles were discovered **D** a frog, *Oreolalaxrhodostigmatus* Hu & Fei, 1979 **E** a running individual of *Qianotrechuscongcongae* sp. nov.

Figures 1 , 3B, E , 6 , 7 , 8

#### Type material.

Holotype male, cave Shigao Dong, Hexi, Nanchuan, Chongqing, 28.82° N, 107.32°E, 729 m, 2021-IV-13, leg. Yi Zhao, Xinyang Jia and Mingyi Tian, in SCAU.

#### Diagnosis.

A small, stout cave trechine, semi-aphaenopsian, with a brown body, rather short appendages and broadly tumid prothorax.

#### Description.

Length: 5.5–5.8 mm, width: 1.9 mm. Habitus as in Figure 6.

Body brown, palps, antennae and tarsi yellow. Surface and underside smooth and glabrous. Microsculpture: isodiametric meshes on head, transversal meshes or striate on pronotum and elytra.

Head (Figs 3B, 6) moderately elongate, longer than wide excluding mandibles, HLl/HW = 1.4; genae moderately convex, widest near head mid-length; frons and vertex moderately convex; frontal furrows nearly parallel-sided, but slightly and shortly convergent posteriorly, ending at widest point of head; anterior and posterior supraorbital pores present, located at about middle and basal 1/5 of head excluding mandibles; clypeus 4-setose, labrum transverse, faintly bisinuate at the front margin, 6-setose; right mandibular teeth bidentate; mentum completely fused with submentum, 2-setose, tooth short and pointed at apex, much shorter than the lateral lobes; ligula fused with paraglossae, 8-setose at apex; palps thin and slender, the 2^nd^ labial palpomere about 0.9 times as long as 3^rd^; 3^rd^ maxillary palpomere as long as 4^th^; suborbital pores close to neck; antennae thin, filiform, extending to about middle of elytra; 3^rd^ antennomere longest, about twice as long as scape; relative length of each antennomere compared with scape in the holotype as follows: 1^st^ (1.0), 2^nd^ (1.1), 3^rd^ (1.9), 4^th^ (1.7), 5^th^ (1.6), 6^th^ (1.4), 7^th^ (1.2), 8^th^ (1.2), 9^th^ (1.0), 10^th^ (1.0) and 11^th^ (1. 4).

Prothorax quadrate, as long as wide, widest behind middle; as long as head excluding mandibles, as wide as pronotum (Fig. 7A). Pronotum slightly wider than head, PnW/HW = 1.2, widest at about apical 1/4, lateral margins finely bordered throughout, suddenly narrowed before hind angles; base and front unbordered, the former narrower than the latter; anterior latero-marginal pores located at apical 1/5 and the posterior pores in front of hind angles. Scutellum small.

Elytra (Fig. 6, 3E) stout, longer than wide, EL/EW = 1.6, wider than pronotum, EW/PW = 2.1, and much longer than fore body including mandibles; lateral margins ciliate throughout; disc strongly convex, striae shallow, easily traceable though more or less reduced, intervals flat; anterior and posterior dorsal pores of the 3^rd^ striae at about basal 1/6 and middle of elytra, respectively, preapical pore at about apical 1/7 of elytra, much closer to elytral suture than to apical margin; only an apical pore present, the anguloapical pore absent.

Legs moderately long and densely pubescent; fore and middle tibiae longitudinally grooved externally, whereas simple in hind tibiae; the 1^st^ and 2^nd^ protarsomeres dilated and spurred inwards at apex in male.

Ventrites IV–VI, each with a pair of paramedial setae, ventrite VII bisetose in male. Male genitalia (Fig. 7C, D): median lobe of aedeagus well-sclerotised, long, and elongate, slightly arcuate at median portion, gradually narrowed toward apex which is bluntly obtuse; basal opening large, with a large sagittal aileron; inner sac provided with a thick and long copulatory piece which is about 2/9 as long as aedeagus; in dorsal view, apical lobe longer than wide, gradually narrowed toward apex which is broadly rounded; parameres well developed, shorter than median lobe, each armed with 4 and 5 long setae at apex.

**Female.** unknown.

#### Remarks.

The genus *Qianotrechus* Uéno, 2000 is comprised of three semi-aphaenosian trechine species and one subspecies (Fig. 1): *Q.magnicollis* Uéno, 2000, *Q.tenuicollistenuicollis* Uéno, 2000, and *Q.tenuicollischeni* Uéno, 2003 from Suiyang County and *Q.laevis* Uéno, 2000 from Zheng’an County, Zunyi Shi, Guizhou Province (Uéno 2000, 2003). Another species, *Q.fani* Uéno, 2003, has been reported from Gulin County, Luzhou Shi, Sichuan Province, but it is not a *Qianotrechus* and so has been transferred into the genus *Uenoaphaenops* Tian & He, 2020 (Tian and He 2020).

*Qianotrechuscongcongae* sp. nov. is closely similar to *Q.laevis* from Zheng’an County of northeastern Guizhou Province (Uéno 2000). The locality of the latter species (Mawan Dong) is about 30 km in a straight line from Shigao Dong in Nanchuan, Chongqing. However, *Q.congcongae* is different from *Q.laevis* in having a stouter body (more elongated in *Q.laevis*), a broader prothorax with propleura widely visible from above (narrowly visible in *Q.laevis*) (Fig. 7A, B), and the pronotum evidently narrowed near the base, which is distinctly sinuate before posterior latero-marginal setae (only slightly sinuate in *Q.laevis*). In addition, the median lobe of the aedeagus is straight and widened at apex (slightly sinuate and narrowed in *Q.laevis*) (Fig. 7C–F).

#### Etymology.

The name of this beautiful species is in honour of “Xiao Cong”, a nickname of Ms Jia Liu, an outstanding and leading caver in the Chongqing Cave Exploration Team, Chongqing, for her kind assistance in our collecting trips in Chongqing.

#### Distribution.

China (Chongqing). Known only from the limestone cave in Shigao Dong, Nanchuan (Fig. 1). Shigao Dong is located about 0.5 km from Hexi Zhen (Nanchuan) in a straight line to the south. The cave, which is near a small road and just behind a farm house, has a large entrance (Fig. 8A). There is an underground river inside the cave. The main passage is about 100 m from the entrance. At first, we reached as far as 300 m along the right passage of the case, which is huge, several dozen metres high and wide (Fig. 8B), but without any finding specimens. Later, we came back to the cave and went explored the left passage, where we observed two beetles running on the wet ground near a small pool, about 160 m from the cave’s entrance (Fig. 8C, E). Apart from *Qianotrechuscongcongae* sp. nov., three species of millipedes, a dipluran, and a troglophilic frog (*Oreolalaxrhodostigmatus* Hu & Fei, 1979) were also observed in this cave (Fig. 8D).

### Qianlongius

Taxon classificationAnimaliaColeopteraCarabidae

Tian & Jia gen. nov.

1D2E586B-FA76-58EE-887F-B411B764CF32

http://zoobank.org/5A4CC615-25EA-4D8F-95E7-F6A6B6A12305/

#### Type species.

*Qianlongiuszhoui* Tian & Jia, sp. nov. (Qianlong Dong cave, Songtao, Guizhou).

#### Generic characteristics.

Large for cavernicolous trechines, semi-aphaenopsian, but more or less pigmented; body stout, appendages moderate long; head and pronotum smooth and glabrous, elytra wholly pubescent; moderately shining. Head subquadrate, distinctly longer than wide excluding mandibles, nearly parallel-sided; frontal furrows sub-parallel-sided, 2 supra-orbital pores present; mandible thin and sharp at apices, right mandible tridentate; labial suture well-marked; mentum bisetose, slightly shallow at base; submentum with a row of 6 setae; palps thin and elongated, the 2^nd^ labial palpomere without additional setae apart from the 2 setae on inner margin; antennae thin and long, extending almost to apices of elytra, scape as long as pedicel. Prothorax slightly tumid at sides and visible from above; prothorax longer than wide, slightly shorter than head excluding mandibles, widest at about basal 1/3; pronotum subquadrate, evidently longer than wide, wider than head, slightly narrower than prothorax; 2 pairs of latero-marginal setae present, both front and hind angles broadly obtuse. Elytra stout, but longer than fore body including mandibles, slightly expanded at sides, widest a little before middle, strongly convex, partly concealed marginal gutter in middle; humeral angles widely rounded, lateral margins smooth, not ciliate, apices almost rounded; elytral striae obliterated though still traceable; 2 dorsal pores present on the 3^rd^ stria, the preapical dorsal pores present; apical striole invisible; humeral group (the 1^st^–4^th^ pores) of marginal umbilicate series not aggregated, 2^nd^, 3^rd^ and 8^th^ pores close to the marginal gutter, 1^st^ pore inwardly and backwardly shifted, 4^th^ pore widely isolated; median group (the 5^th^ and 6^th^ pores) backwardly shifted, close from each other. Protarsomeres not modified in male; the 1^st^ tarsomere much shorter than 2^nd^ to 4^th^ tarsomeres combined in fore legs, whereas as long in middle and hind legs. Ventrite VII with 2 pairs of apical setae in male. Male genitalia short and thick, moderately sclerotized.

#### Remarks.

The position of this new genus within the tribe Trechini remains unclear. However, *Qianlongius* gen. nov. is, on the first sight, more or less similar in body shape and colouration to *Guizhaphaenopsodes* Tian & X. Huang, 2020 (from Tangle Dong cave in Jishou, western Hunan), but these genera belong to different lineages on account of the following differences in important charateristics. First of all, *Qianlongius* is more aphaenopsian than *Guizhaphaenopsodes* due to its thinner body with a more elongated head, distinctly reduced frontal furrows, propleura tumid and visible from above, and thinner antennae reaching the elytral apices. Second, the right mandibular tooth is tridentate, and mentum and submentum are not fused in *Qianlongius*, versus bidentate right mandibular tooth, and completely fused mentum and submentum in *Guizhaphaenopsodes*. Third, the male protarsi of *Qianlongius* are simple, while the first protarsomere is modified in *Guizhaphaenopsodes*. Fourth, ventrite VII is 4-setose in the male of *Qianlongius*, instead of bisetose in *Guizhaphaenopsodes*. And fifth, the male genitalia are quite large and moderately elongated in *Qianlongius*, while small and very short in *Guizhaphaenopsodes*.

#### Etymology.

Refering to Qianlong Dong cave, locality of the type species *Qianlongiuszhoui* sp. nov. Gender masculine.

#### Generic range.

China (Guizhou). Known only from cave Qianlong Dong (Fig. 1).

### Qianlongius zhoui

Taxon classificationAnimaliaColeopteraCarabidae

Tian & Jia sp. nov.

5235AD4A-77B4-59BE-B209-484F7344EE82

http://zoobank.org/602A3633-B600-4BC4-B6FD-649C01F0CDE0

**Figure 9.**
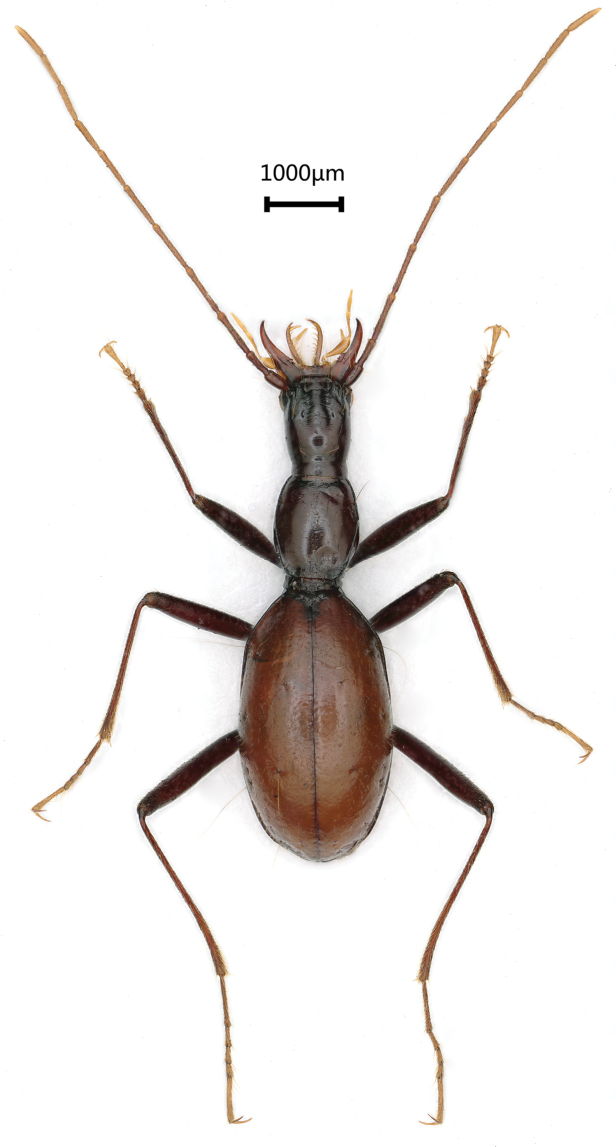
*Qianlongiuszhoui* gen. nov. and sp. nov., habitus, holotype male.

**Figure 10.**
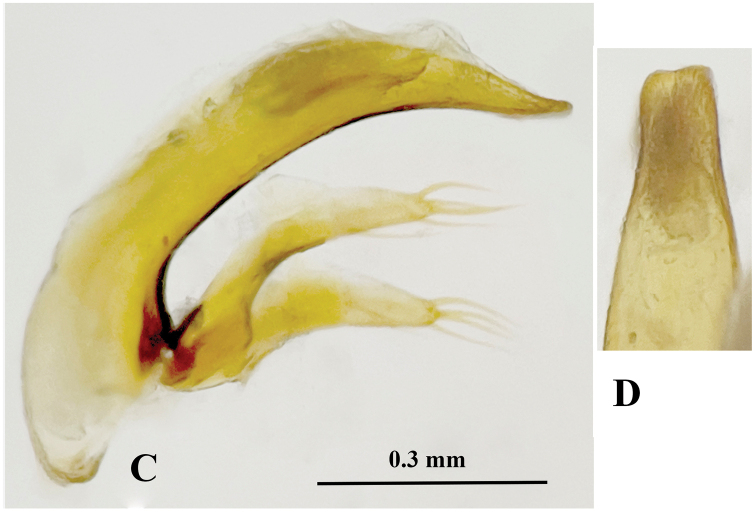
Male genitalia of *Xianlongiuszhoui* gen. nov. and sp. nov. **A** median lobe and parameres, lateral view **B** apical lobe, dorsal view.

**Figure 11.**
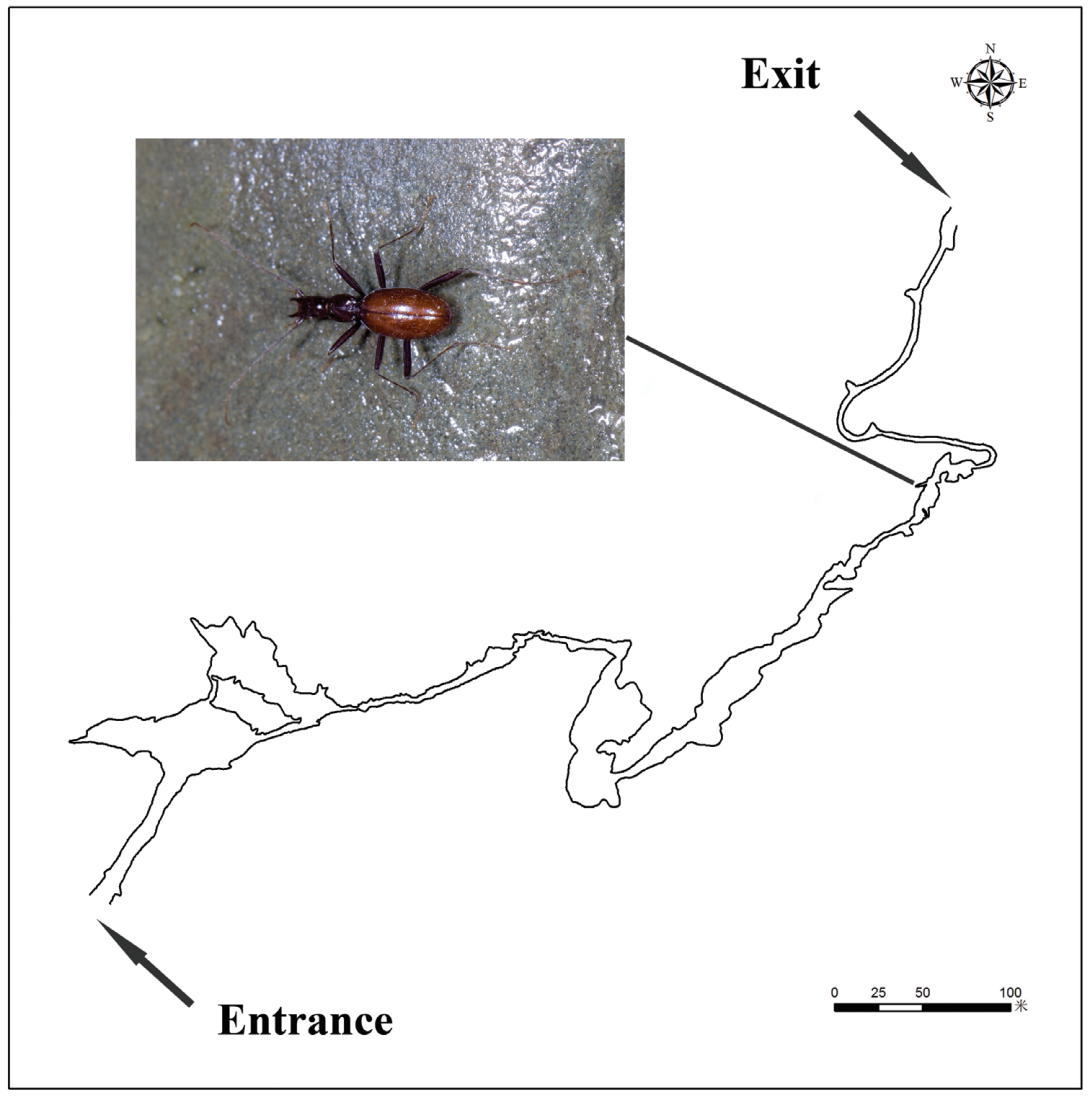
Map of Qianlong Dong, to show where the exemplars of *Qianlongiuszhoui* gen. nov. and sp. nov. were discovered (map courtesy of Mr Wenlong Zhou).

**Figure 12.**
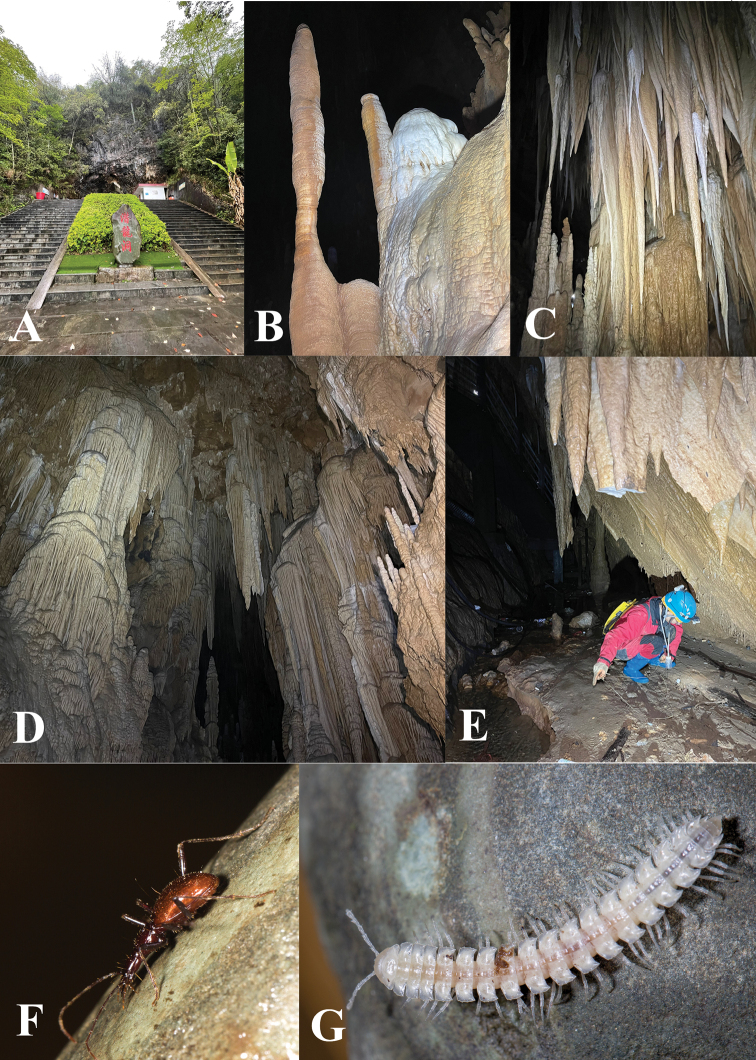
Cave Qianlong Dong **A** entrance **B–D** beautiful speleothems inside the cave **E** the locality of *Qianlongiuszhoui* gen. nov. and sp. nov. **F** a running individual of *Q.zhoui* gen. nov. and sp. nov. **G** a millipede, *Epanerchodus* sp.

Figures 1 , 3C, F , 9 , 10 , 11 , 12

#### Type material.

Holotype male, cave Qianlong Dong, Qianlong, Wuluo, Songtao, Tongren, Guizhou Province, 28.16° N, 108.84°E, 908 m, 2021-IV-2, leg. Yi Zhao, Xinyang Jia and Mingyi Tian, in SCAU. Paratype: 1 male, ibid.

#### Diagnosis.

A medium-sized, semi-aphaenopsian species, with a rather stout body though both head and pronotum longer than wide, head and pronotum dark brown, elytra reddish-brown, fore body including mandibles slightly shorter than elytra, antennae extending to apices of elytra, propleura visible from above.

#### Description.

Length: 6.5–7.0 mm, width: 2.0–2.1 mm. Habitus as in Figure 9. Head, pronotum, undersides of head and thorax, ventrites, femora and tibiae dark brown, elytra, labrum and mandibles reddish-brown, palps, antennae and tarsi yellow. Head smooth, with a few short setae, 1 or 2 on dorsal surface, while 2 on genae; pronotum and elytra with short pubescence, prosternum bisetose; abdominal ventrites wholly pubescent. Microsculpture: isodiametric meshes on neck, transversal striate on frons, vertex, pronotum and elytra.

Head moderately elongate, longer than wide, HLm/HW = 2.0–2.4, HLl/HW = 1.5–1.7; nearly parallel-sided, widest at about middle of head excluding mandibles; frons and vertex convex; frontal furrows wide and incomplete, more or less parallel-sided, but slightly and shortly convergent backwards, ending about middle of head; anterior and posterior supraorbital pores located at about 4/7 and ¼ of head from labrum to neck; clypeus 6-setose, labrum transverse, faintly bisinuate in the front margin, 6-setose; mandibles developed and moderately curved at apices; mentum tooth short but sharp at apex, bifid at tip, slightly shorter than the lateral lobes; ligula fused with paraglossae, 8-setose; palps thin and slender, the 2^nd^ labial palpomere about 1.2 times as long as 3^rd^; 3^rd^ maxillary palpomere 1.1times as long as 4^th^; suborbital pores absent (Fig. 3C); antennae thin, filiform, pubescent from pedicle to 11^th^ antennomeres, 3^rd^–5^th^ antennomeres the longest, each almost twice as long as scape; relative length of each antennomere compared with scape in the holotype as follows: 1^st^ (1.0), 2^nd^ (1.1), 3^rd^ (2.0), 4^th^ (2.1), 5^th^ (2.1), 6^th^ (1.9), 7^th^ (1.8), 8^th^ (1.6), 9^th^ (1.6), 10^th^ (1.4) and 11^th^ (1. 6). Prothorax slightly longer than wide, PrL/PrW = 1.1–1.3; Pronotum longer than wide, PnL/PnW = 1.1–1.2; slightly narrower than prothorax, PrW/PnW = 1.0–1.1; wider than head, PnW/HW = 1.2, but shorter than head excluding mandibles, PnL/HLl = 0.9; widest at about apical 3/5, lateral margins gently expanded, narrowly and evenly bordered throughout, shortly reflexed near hind angles which are widely obtuse; base and front unbordered, almost straight, the former slightly narrower than the latter; anterior latero-marginal pores located at apical quarter and the posterior pores a little before hind angles; frontal and basal transverse impressions faint; middle line well marked; disc moderately convex. Scutellum small. Elytra (Fig. 3F) much longer than wide, EL/EW = 1.9, wider than pronotum, EW/PW = 1.8–1.9, and longer than fore body including mandibles EL/(HLm+PnL) = 1.1; unbordered at base; disc strongly convex, intervals flat; basal pore at side of scutellum, located against 3^rd^ stria; anterior and posterior dorsal pores of the 3^rd^ striae at about 1/3 and 2/3 of elytra from base, respectively; preapical pore at about apical 1/9 of elytra, closer to elytral suture than to apical margin; only an apical pore present; pore 7 distant from elytral marginal gutter.

Legs moderately long and densely pubescent; fore tibiae longitudinally grooved externally, whereas middle and hind tibiae simple.

Ventrites IV–VI each with a pair of paramedial setae. Ventrite VII with 2 setae in male.

Male genitalia (Fig. 10): median lobe of aedeagus rather short but stout, slightly arcuate at median portion, gradually narrowed toward apex, which is more or less reflexed; basal opening large, without a sagittal aileron; inner sac provided with a thick and long copulatory piece which is about 2/9 as long as aedeagus; in dorsal view, apical lobe longer than wide, apical margin broad, but emarginate at middle; parameres well developed and elongated, but much shorter than median lobe, each armed with 4 long setae at apex and anotherseta at subapex.

**Female.** unknown.

#### Etymology.

In honour of Mr Wenlong Zhou, an active speleologist (Guizhou Institute of Mountainous Region Resources, Guiyang) to thank him for supporting our survey in Qianlong Dong.

#### Distribution.

China (Hunan). Known only from limestone cave Qianlong Dong (Fig. 1).

Qianlong Dong is a show cave located at Qianlong village, Wuluo Zhen, Songtao Miao Autonomous County, northeastern Guizhou. This beautiful cave is 1481 m long and with many wonderful speleothems (Barbary et al. 2011) (Fig. 11, 12B–D). Some parts inside the cave have natural conditions, although almost the entire cave has been developed for touristic purposes. Both specimens of this new species were discovered in the innermost part of the cave along the creek, not far from the artificial exit tunnel; one beetle was running on the ground and the other was found under a stone (Fig. 12E, F). Other cave animals observed in Qianlong Dong were: a millipede (*Epanerchodus* sp.; Fig. 12G), springtails, crickets, and a harvestman.

## Supplementary Material

XML Treatment for Wulongius

XML Treatment for Wulongius qilinger

XML Treatment for Qianotrechus congcongae

XML Treatment for Qianlongius

XML Treatment for Qianlongius zhoui
